# Knockdown of a cellulose synthase gene *BoiCesA* affects the leaf anatomy, cellulose content and salt tolerance in broccoli

**DOI:** 10.1038/srep41397

**Published:** 2017-02-07

**Authors:** Shuangtao Li, Lei Zhang, Ying Wang, Fengfeng Xu, Mengyun Liu, Peng Lin, Shuxin Ren, Rui Ma, Yang-Dong Guo

**Affiliations:** 1 College of Horticulture, China Agricultural University, 100193, Beijing, China; 2 Horticulture Research Institute, Shanghai Academy Agricultural Sciences, Shanghai 201403, China; 3 School of Agriculture, Virginia State University, PO Box 9061, Petersburg, VA23806, USA; 4 Agro-Biotechnology Research Institute, Jilin Academy of Agricultural Sciences, Changchun 130033, China

## Abstract

Cellulose is the major component of cell wall materials. A 300 bp specific fragment from the cDNA fragment was chosen to insert into vector pFGC1008 at forward and reverse orientations to construct the recombinant RNAi vector. Knockdown of *BoiCesA* caused “dwarf” phenotype with smaller leaves and a loss of the content of cellulose. Moreover, RT-PCR analysis confirmed that the expression of the RNAi apparatus could repress expression of the *CesA* gene. Meanwhile, examination of the leaves from the T3 of RNAi transformants indicated reduction of cell expansion in vascular bundles, particularly on their abaxial surface. The proline and soluble sugar content increased contrarily. Under the salt stress, the T3 of RNAi plants showed significant higher resistance. The expression levels of some salt tolerance related genes (*BoiProH, BoiPIP2;2, BoiPIP2;3*) were significantly changed in T3 of RNAi plants. The results showed that the hairpin structure of *CesA* specific fragment inhibited the endogenous gene expression and it was proved that the cDNA fragment was relevant to the cellulose biosynthesis. Moreover, modulation cellulose synthesis probably was an important influencing factor in polysaccharide metabolism and adaptations of plants to stresses. This will provide technological possibilities for the further study of modulation of the cellulose content of crops.

Dietary fiber is believed to protect against a series of diseases^1^. Most of dietary fiber is from cell walls of plants. Cellulose, an essential component of both primary and secondary cell walls of high plants^2^, is composed of (1 → 4)-β-D-glucan chains^3^. The first plants cellulose synthase (*CesA*) gene was cloned in 1996^4^ and the isolation of cellulose synthase complex was difficult^5^. The discovery of acotton gene suspected to encode *CesA*brought the field of plant cell wall biogenesis into the genomic era^6^. Many *CesA*and*CesA*-like (*Csl*) genes have been isolated in plants by far^7^,^8^,^9^,^10^.

Sequence analyses of the *CesA* genes indicated that they encoded family II glycosyl transferases^11^,^12^. These enzymes contained two domains designated A and B. Domain A contained the D … D motif common to all family II glycosyltransferases while domain B carried an additional conserved D residue as well as the QxxRW motif^11^,^12^,^13^. Structural evidence of family II and other glycosyl transferases suggested that the A domain binded the nucleotide sugar and the B domain binded the acceptor substrate, together forming a viable catalytic center^14^,^15^. Moreover, there were two N-terminal putative zinc finger domains in the *CesA* proteins, and might play a key role in the dimerization of the *CesA* catalytic subunits and the rosette assembly^16^. A series of mutants can be used to analysis the function of different *CesA* genes. For example, temperature-sensitive *root-tip swelling* mutant (*rsw*1) of *Arabidopsis* showed a decline of cell wall cellulose content and a dwarf phenotype^17^. The defective *AtCesA6* mutant (*prc1*) presented dwarf-hypocotyl^18^. The *irx1* and *irx3* mutant, displaying a phenotype of collapsed mature xylem cells and reduced content of secondary cell wall cellulose, were determined to be *CesA* homologues^19^,^20^,^21^. Moreover, the expression levels of *CesA* RNA and accumulation of cellulose content have been evaluated in tobacco^22^.

By antisense expression of different potato *CesA* clones, the cellulose content of tuber cell walls dropped to 40% of the control plant and the recombined constructs are efficient to control the cellulose synthesis^23^. Currently, we focused on using RNAi to changes cellulose levels and anatomic characteristics of broccoli. The correlation of anatomic changes and plant physiological character was also discussed. The aim of present study is mainly regulate the content of cellulose, so as to improve the quality of vegetables.

## Materials and Method

### Amplification of the broccoli *CesA* fragment

Total RNA was extracted from young leaves of broccoli by using the procedure of phenol-guanidine isothiocyanate (Trizol, invitrogen). The RNA was then used as template to synthesize the cDNA.

We used the following sequences: cotton *GhCesA* (U28583 and U28584^4^), *ArabidopsisAtCesA1* (AAC39334.1^17^), *Acetobacter*BcsA (M37202), *Acetobacter*AcsA (X54676) as described in ref. 24, primers were designed according to the *CesA* genes in cotton, *Arabidopsis, Acetobacter xylinum*. Fragments of cDNAs from broccoli, designated *CesA*-a, *CesA*-b, *CesA*-c, *CesA*-d, *CesA*-e were amplified by PCR from 1 μL of the cDNA reaction mixture with primer combinations, as follows: *CesA*-a, 5′ primer 5′-CTCATCTATGTTTCTCGTGA-3′and 3′ primer 5′-GCATCTTGAACCCAGTAA-3′; *CesA*-b, 5′ primer 5′-TAACAGGGA-GACTTATCTTGACCG-3′ and 3′ primer 5′-GGAACTGGACATAGCACACTT-3′; *CesA*-c, 5′ primer 5′-GGAAAGATGGAACTCAGTG -3′ and 3′ primer 5′-CGTTACAAGAGGAG-GCTC-3′; *CesA*-d, 5′ primer 5′-CGTGTTGAAGATGGAGA-3′ and 3′ primer 5′-AGATTG-TGTATCAGGCGTGC-3′; *CesA*-e,5′ primer 5′-AGTGTAAGAAAGCGTTTTGGTCA-3′ and 3′ primer 5′-CAATGACCCAGAACTGCTCG-3′. PCR amplification was performed with standard PCR buffer, 50 ng of both *CesA* primers, and 2.5 units of Taq polymerase (TAKAR-A). The BLAST programs, Clustal analysis and multiple alignment of the DNAMAN program package, were used to analyze the homology of cDNA sequences.

Total RNA was extracted from various tissues of four-week-old wild type plants. Quantitative real-time PCR was carried out in ABI7500 system with the SYBR Premix Ex Taq^TM^ kit (TAKARA, Japan). The primer pairs were used for the experiment as follows: *BoiCesA* primers, F (5′-CGTGTTGAAGGAGATGGAGA-3′), R (5′-AGATTGTGTATCAGGCGT-GC-3′). *Actin*gene(AF044573) primers, ActF (5′-TGGGATGAACCAGAAGGATGC-3′) and ActR(5′-TGGC-GTAAAGGGAGAGGACA-3′)30 cycles. The specific of primer pairs was checked (Fig. S1). Each experiment was replicated at least three times.

### Construction of RNAi vector

A 300-bp class-specific region was amplified to construct the recombined RNAi vector pFGC*CesA* which used the primers RiF containing *Bam*HI and *Spe*I sites(F1-AAAGGATCCAAAGATGGAACTCAGT; R1-GAAACTAGTGCACAGATTGTGTATCAG) and RiR containing *Asc*I and *Swa*I sites (F2-AAGGCGCGCCATTGTGTATCAGGC; R2-GGGATTTAAATGAGAGGAAAGATGG). The *BoiCesA* sense and antisense fragments were inserted into pFGC1008 to construct the recombined vector. The selection of specific cDNA fragment referred to the method that usedvirus-induced gene silencing which published in the Plant Cell^25^. The vector was transferred into A*grobacterium tumefaciens*stain EHA105 by the freeze-thaw method^26^.

### Genetic transformation of broccoli

The broccoli variety 05-33-105 was used for transformation. It was implemented with *Agrobacterium tumefaciens* stain EHA105 harboring pFGC*CesA* constructs and using plasmid pFGC1008 as control. The recombinant plasmid includes the *HPTII* coding region which was used as a selectable marker (conferring hygromycin resistance).

A hygromycin sensitivity test was performed using cotyledon and hypocotyl explants from seven-day-old seedling^27^. Hypocotyl and cotyledon explants were pre-incubated on the shoot induction medium (MS medium containing 2 mg/L ZT and 0.01 mg/L IAA) for two days in darkness. The incubated explants were immersed into the *Agrobacterium tumefaciens* solution for 4–8 min (to the hypocotyls and cotyledons) with gentle shaking. The explants were then transferred on the co-cultivation media (MS medium containing 2 mg/L ZT, 0.01 mg/L IAA and 100 μM AS). After co-cultivation for two days in darkness, the explants were transferred to the same basal medium which was supplemented with 350 mg/L carbenicillinand cultured for seven days. Then the explants were transferred on the selection medium which was supplemented with hygromycin at 4 mg/L and carbencillin at 200 mg/L for another 4–6 weeks to induce shoots. When the shoots emerged, they were subjected to transfer to another medium (MS medium containing 0.5 mg/L NAA and 5 mg/L hygromycin) for root induction. Finally the regenerated plants were transferred to soil. After being vernalized, the seeds of progeny were obtained. The transgenic lines (T3 lines) were used for further experiments.

### Southern blotting

The genomic DNA was extracted from control and transgenic plants, using *Bam*HI to digest the DNA. The DNA was transferred and cross-linked onto a nylon membrane. The selectable hygromycin phosphotransferase gene (*HPT*II) was labeled by PCR for hybridization (Dig Easy Hyb). Then the membrane was washed with different concentration of SSC. At last the membrane was exposed to X-ray^28^. The primers of *HPT*II gene is 5′-CGTGTTGAAGGAGAT-GGAGA-3′ and 5′-AGATTGTGTATCAGGCGTGC-3′.

### Microscopy analysis

For light microscopy, developed leaves were used to prepare sections by microtome and it were stained with safranin-fast green, then observed and photographed under a light microscope^29^. For scanning electron microscopy (SEM), the methods are detailed by Yu *et al*.^30^. Small leaf tissues were fixed with 2.5% buffered glutaraldehyde. Then, it was transferred to 1% osmium tetroxide fixative and dehydrated in an ethyl alcohol series from 30 to 100%. The important step was critical point dried and gold coated transmission electron microscopy (TEM) sampling and preparation were carried out as described in the standard procedure^31^.

### Measurement of carbohydrate

Cell walls were prepared based on previous methods^32^,^33^. Briefly, using the phenol-methanol to eliminate lipid and protein from the sample and extracting with ethanol and drying, the dried cell wall materials were used to analyze the cellulose content^34^.

The measurement of pectin content was operated as described in papers^35^,^36^. Shortlythe sample powder with hot absolute ethanol was heated and then centrifuged at 10,000 rpm for 10 min. Alcohol insoluble solids (AIS) were obtained and the concentrated sulfuric acid was used to dissolve AIS. The mixture was transferred into a 25 ml volumetric flask. Then sample solution was added to sodium tetraborate. Color development following addition of *m*-hydroxydiphenyl, the galacturonic acid was gained that was equal to total pectin content.

### Reverse Transcription PCR method

Leaves were picked from T3 of RNAi plants and ground to the fine powder in liquid nitrogen, total RNA was extracted according to the method described by the scription of Trizol. Ten μg of RNA was used for cDNA synthesis with oligo (DT) _18_ as the primer and 1 μL of cDNA was applied in the PCR reaction. The cycle numbers and transcript levels were optimized.

### Proline and soluble sugar content determination

Two independent transgenic lines were selected to measure the proline and soluble sugar content. The measurement of proline content in leaves was prepared according to the method reported by Troll and Lindsley^37^. The content of soluble sugar was then measured^38^.

### Evaluation of NaCl to tolerance for T3 of RNAi plants

The control plants and RNAi plants were kept in a chamber with normal growth condition as 16-h-light/8-h-dark cycle, 23 ± 1 °C, with 60% relative humidity. For NaCl treatments, the four-week-old potted plants were treated with 250 mM NaCl for 3 weeks.

### The assay of antioxidant enzymes

Superoxide dismutase (SOD; EC 1.15.1.1) activity was measured based on its ability to inhibit the photochemical reduction of Nitroblue tetrazolium^39^. Peroxidase (POD; EC 1.11.1.7) activity was measured at 25 °C by monitoring the increase in absorbance at 470 nm^40^. Catalase (CAT; EC 1.11.1.6) activity was measured at 25 °C by the absorbance decrease at 240 nm due to the H_2_O_2_ decomposition^41^. Ascorbate peroxidase (APX; EC 1.11.1.11) activity was determined by monitoring the decrease inabsorbance of ascorbic acid at 290 nm^42^.

### Quantitative RT-PCR analysis of *BoiProDH, BoiPIP2;2* and *BoiPIP2;3*

The expressions of *BoiProDH, BoiPIP2;2* and *BoiPIP2;3* in WT and T3 of RNAi plants were analyzed by real-time quantitative reverse transcriptase using the fluorescent intercalating dye SYBRGreen in a detection system. The primer pairs were used for the experiment as follows: *BoiProDH* primers, F 5′-CAAGAAGCCGAGAAGGAA-3′, R 5′-CCAGAGTCAGCGTTATGT-3′. *BoiPIP2;2* primers, F 5′-TGTTTGGGTGCGATATGTGGAGTT-3′, R 5′-GTGGCGGAGAAGACGGTGTAG-3′. *BoiPIP2;3* primers, F 5′-AAGGAAGGTATCGTTGGTTA-3′, R 5′-AGTCTCGGGCATTTCTTT-3′. Actin gene (AF044573) primers were same as mentioned above.

### Statistical analysis

Statistical procedures were carried out with the software package SPSS11.0, Differences among treatments were analyzed taking P < 0.05 as significance according to Duncan’s multiple range test. The relative estimate of the amount of cDNA in broccoli leaves was obtained by Image J software.

## Results

### Molecular cloning and comparative sequences analysis of *BoiCesA* cDNA

**Figure 1:**
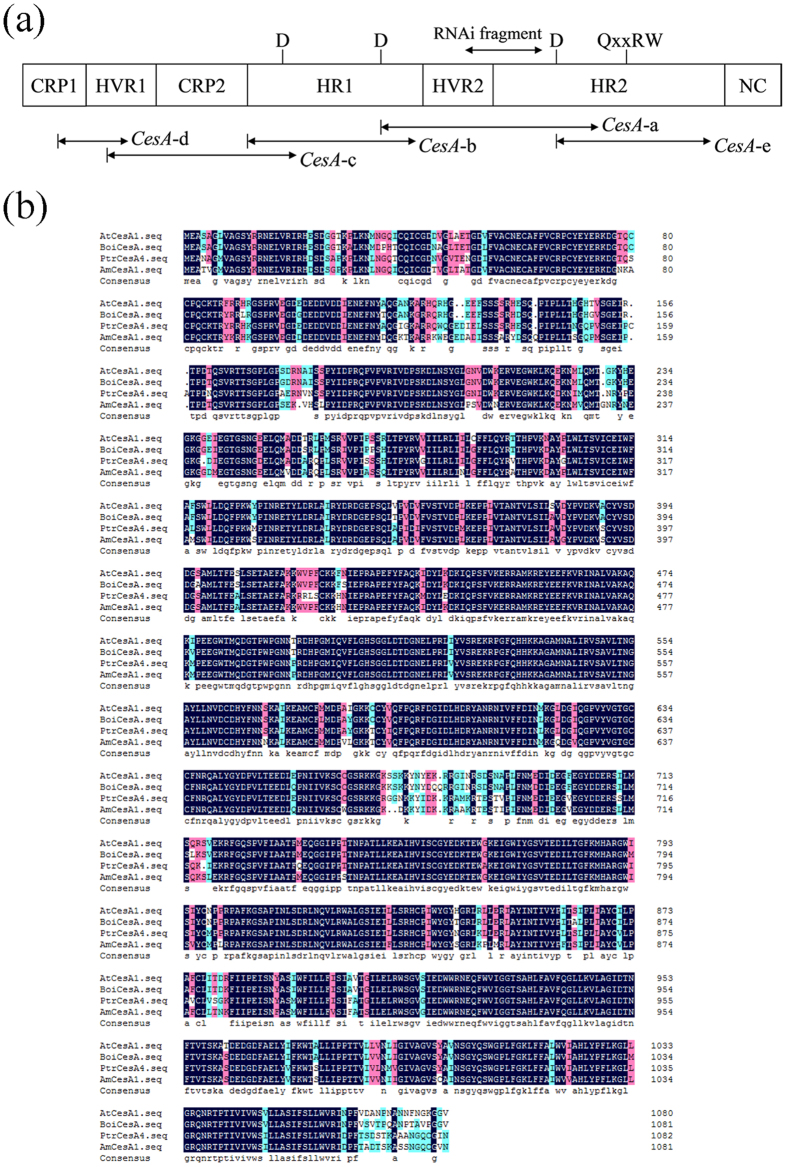
The construct of *BoiCesA* gene and the amino acid sequence alignments of the *Brassica oleracea* L. cellulose synthase *BoiCesA* with *CesA* genes of other plants. (**a**) Positions of the five cDNAs from broccoli were shown in relation to the regions of plant *CesA* genes. CRP: conserved plant-specific region; HVR: hypervariable plant-specific region; HR: homologous region of all *CesA*genes; NC: no obvious conservation; RNAi fragment: the target sequences of RNAi. (**b**) Amino acid sequence alignments of *BoiCesA* with the corresponding sequences of *CesA* from *Arabidopsis (AtCesA1*), *Populustremuloides PtrCesA4* and *Acacia mangiumAmCesA1*.

Five cDNA fragments from the *CesA* gene of broccoli were amplified by standard RT-PCR. Their positions based on the cell wall cellulose biosynthesis gene were shown (Fig. 1a)^4^, as described by Delmer^5^. The nucleotide sequences of cDNAs *CesA-a, CesA-b, CesA-c, CesA-d* and *CesA-e* were identical where they overlap each other. The cDNAs described the sequences of the same *CesA* gene. Based on the results of sequencing and assembly, a 3252 bp of the *CesA* cDNA fragment was identified from broccoli, its corresponding deduced amino acid contained D, D, D (aspartic acid residues) and QXXRW motif which was located at the catalytic site. The sequence of the cDNA was compared with the corresponding sequences of the *Populus tremuloides PtrCesA4* gene, *Acacia mangium AmCesA1* gene and the *Arabidopsis AtCesA1 (rsw1*) gene (Fig. 1b). Sequence analysis shows that the cDNA fragment designated *BoiCesA*is a member of *CesA* superfamily. It shared 90% identity with *AtCesA1* at the nucleotide level and 94% identity at the protein level.

### Organ-specific expression of *BoiCesA* gene

**Figure 2:**
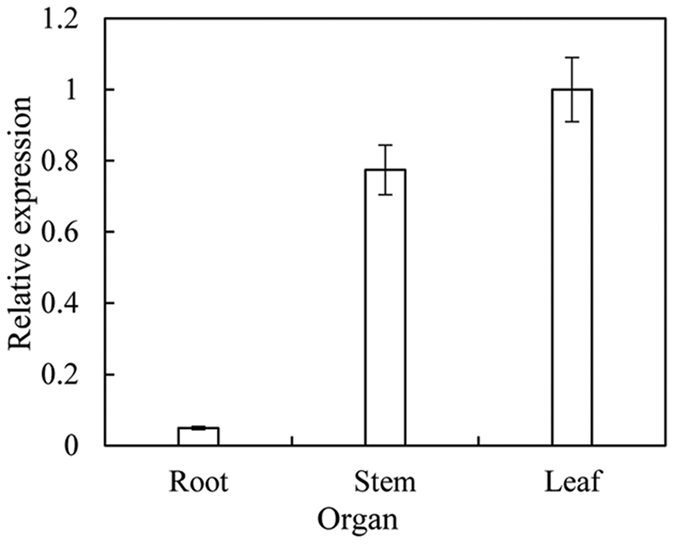
Detection of *BoiCesA* expression level in different organs ofbroccoli plants.

To evaluate the transcript accumulation of *BoiCesA* gene in different organ, the results of quantitative real-time PCR revealed that *BoiCesA* was expressed in various organs of broccoli, including roots, stems and leaves (Fig. 2). Our results showed that the expression level of *BoiCesA* was the highest in leaf organs. There were significant differences (P < 0.05) in the expression level of *BoiCesA* between different organs.

### Regulation of *CesA* gene expression in broccoli

**Figure 3:**
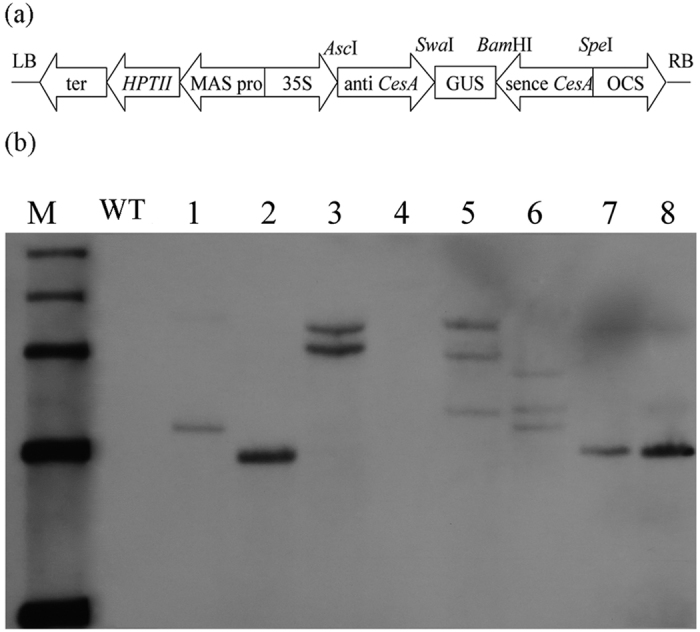
RNAi construct and Southern blotting detection of transgenic plants. (**a**) Schematic diagram of the RNAi construct named pFGC*CesA*. (**b**) Southern blotting detection of transgenic plants. M: Marker DL15000WT: wild-type plants 1–8: transgenic plants.

A recombined RNAi construct pFGC*CesA* was applied to regulate cellulose biosynthesis in broccoli (Fig. 3a). The 300 bp-length sense *BoiCesA* sequence and antisense *BoiCesA* sequence was amplified and inserted into pFGC1008 vector. The T-DNA region of pFGC*CesA* harbored the selectable hygromycin phosphotransferase gene (*HPTII*) for hygromycin resistance. Expression of the hairpin structure was driven by the constitutive CaMV 35S promoter.

### The Southern blotting analysis for transgenic plants

The pFGC1008-*CesA* plasmid was transformed into broccoli and 65 plantlets from hypocotyls and 40 plantlets from cotyledons resistant to hygromycin were obtained. To further confirm that the phenotype of transgenic plants is due to the introduction of RNAi construct, southern blot was done in the control and transgenic plants (Fig. 3b). The wild type plants was used as control that is no band was detected. However, transgenic plants had different hybridization bands which were different size. It suggested that the RNAi construct contained *BoiCesA* gene was random integrated into *Brassica oleracea*. We selected two independent RNAi lines RNAi-2 and RNAi-8 to do further research.

### Transcriptional activity of the *CesA* gene in broccoli

**Figure 4:**
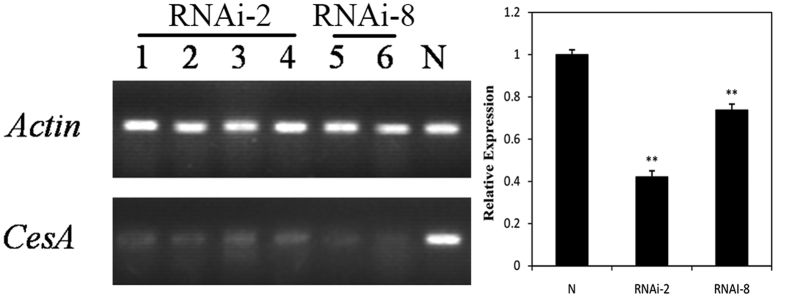
RT-PCR analysis of the T3 of RNAi plants and the control broccoli plants. *Actin*: Agarose gel of RT-PCR products amplified by the primers of *Actin. CesA*: Agarose gel of RT-PCR products amplified by the primers of *BoiCesA.* 1~4: these plants belonged to transgenic line 2 which had been chosen to measure the cellulose content 5~6: these plants belonged to transgenic line 8 which had been chosen to measure the cellulose content. N: The control plants.

In order to evaluate the effect of RNAi on *CesA* gene expression, RT-PCR experiments were performed to study these changed cellulose contents whether related to the expression of *BoiCesA* gene. The constitutive *Actin* gene^43^ applied as the control in this study. It showed that the RT-PCR results with the *BoiCesA* and *Actin* primers in the transgenic plants and control plants (Fig. 4). The amplified product revealed that a reduction of the *BoiCesA* expression in the RNAi plants in relation to the control plants.

### Phenotypes of the RNAi transformed plants

**Figure 5:**
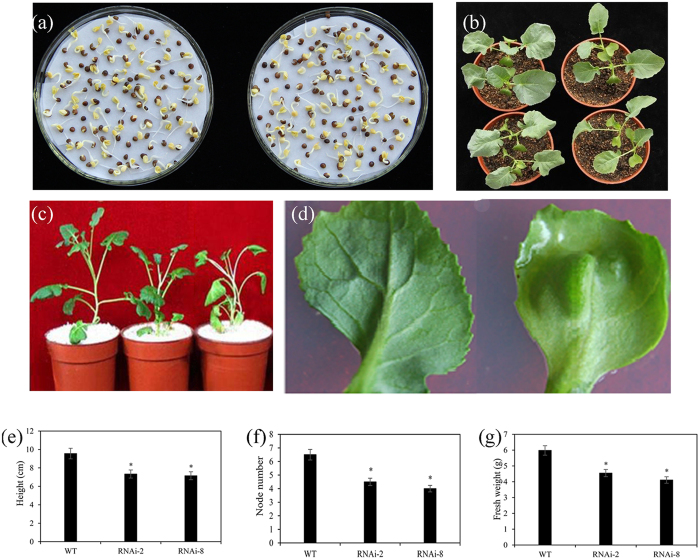
Analysis of phenotype of the broccoli plants. (**a**) The rate of germination between control (left) and transgenic plants (right). (**b**) Growth of control (left) and transgenic plants (right) with three weeks. (**c**) Show left to right is a pFGC1008 plant (control), two RNAi plants. The RNAi plants have obvious change in plant height. (**d**) The underside surface of the leaves are taken from the control plants (left), the RNAi plants (right). The lumps are evident and the texture is crunchy in RNAi plants compared with controls. (**e**) The measurement of height of control and transgenic plants. (**f**) Comparision of node number of control and transgenic plants. (**g**) Analysis of fresh weight for control and transgenic plants. (Values are mean ± SE, *n* = 3, *P < 0.05, **P < *0.01*).

In order to observe the growth of control and knockdown plants, the germination performance of seeds was observed. There is no significant distinction between control and transgenic seeds (Fig. 5a). However transformed plants showed a typical dwarf phenotype (Fig. 5b) and had obvious change in plant height (Fig. 5c). Furthermore, it was evident to find that some surface lumps presented on the abaxial surfaces of the leaves, and the texture was crisp in the RNAi plants (Fig. 5d). The pFGC*CesA* plants were shorter in stature than the control plants (transformed with pFGC1008) (Fig. 5e). Compared with the control plants, the RNAi plants had similar internode length but with less nodes (Fig. 5f). The leaves of the transgenic plants were smaller than those of the control plants, meanwhile the fresh weight decreased relative to that of control plants (Fig. 5g). These phenotypic characteristics had a good agreement with the corresponding observation in tobacco which had been silenced by a plant cellulose synthase gene^25^.

### Anatomic and ultrastructural changes

**Figure 6:**
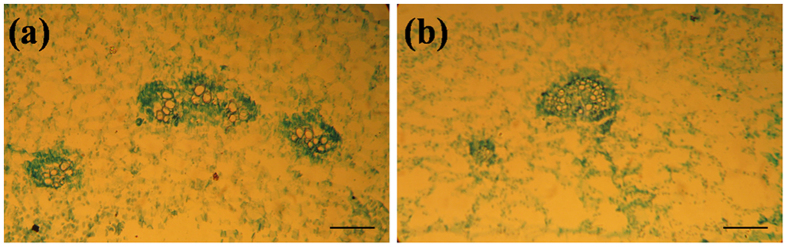
Light microscopic analysis of the control broccoli plants and the T3 of RNAi plants. (**a**) Transverse section of the leaf vein in control plants. (**b**) Transverse section of the leaf vein in the T3 of RNAi plants. All cells of the vascular bundles showed reduced expansion compared with the control plants. Bars indicate 100 μm for (**a**,**b**).

The difference of tissue structure between the T3 of RNAi plants and the control broccoli was surveyed by the light microscopy. The vascular bundles was reduced on the transverse sections located in the elongation zone of leaf veins. Compared with the control plant, the development of lateral veins was not observed in the T3 plant (Fig. 6). Meanwhile, all cells of the vascular bundles of the RNAi plants reduced expansion or elongation but the control plant had the normal leaf veins, compared to control plant, the content of vascular bundles of RNAi plants was approximately 56% (Fig. 6).

**Figure 7:**
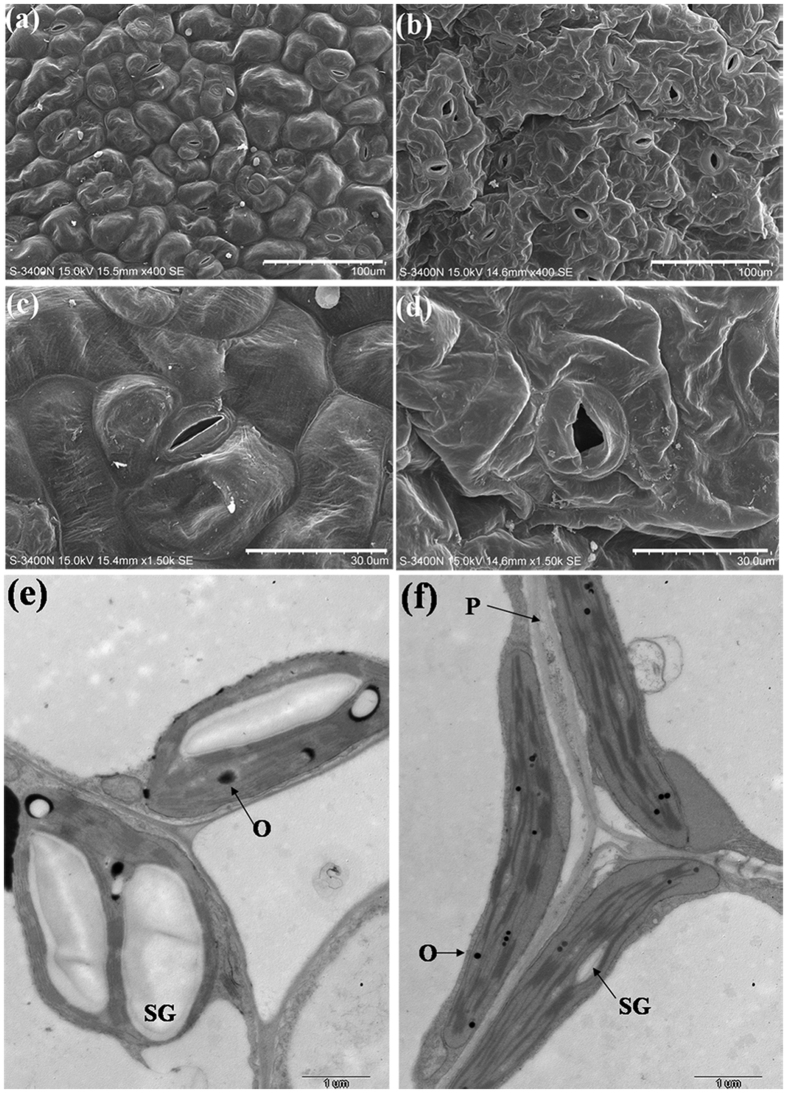
Scanning and transmission electron microscopic analysis of the control broccoli plants and the T3 of RNAi plants. (**a**) Abaxial surface of the control leaves showing smooth epidermal cells. Bar = 100 μm. (**b**) Abaxial surface of the T3 of RNAi leaves showing swollen epidermal cells. Bar = 100 μm. (**c**) Normal stomata of the control leaves with kidney-shaped guard cells. Bar = 30 μm. (**d**) Abnormal stomata of the T3 of RNAi leaves with deformations of guard cells. Bar = 30 μm. (**e**) Normal chloroplast of the control leaves with the regular and inseparable layer of chloroplast grana and stroma, which overflow with starch grains. Bar = 1 μm. (**f**) Chloroplast of the T3 of RNAi leaves shows the numbers and types of starch grain differ from the control plants, numerous osmiophilic globules appear in the T3 plants. Bar = 1 μm. O: osmiophilic globules; P: plasmolysis; SG: starch grain.

Scanning electron microscopy of the leaves from the control plants showed that the abaxial surface were generally smooth, and the epidermal cells were arrayed orderly (Fig. 7a). The stomata of the control plantlets displayed the normal morphology with kidney-shaped guard cells (Fig. 7c). On the contrary, there were many clumps of the epidermal cells along the abaxial surface, especially adjoin to leaf vein in RNAi plants (Fig. 7b). The RNAi and the control leaves also differed in the stomata morphological specificity, the T3 of RNAi leaves presented the abnormal stomata, with guard cells drastically deformed due to the swollen epidermal cells (Fig. 7d). The deformation of guard cells could possibly affect the stomatal function.

Moreover, some significant differences were also observed between the ultrastructure of chloroplasts in the T3 of RNAi and the control leaves. The results of transmission electron microscopy showed that the control cells chloroplasts of mesophyll cells contained the entire double membranes, the regular and inseparable layer of chloroplast grana and stroma, which overflow with starch grains (Fig. 7e). Whereas in the RNAi plants the layer of the slender and spindle-shaped chloroplast grana and stroma were irregular and even disaggregated, but most of all, there was a great difference between the numbers and types of starch grain from the RNAi and the control leaves, furthermore numerous osmiophilic globules appeared in theRNAi plants (Fig. 7f).

### The T3 of RNAi plants have altered cellulose and pectin content

**Figure 8:**
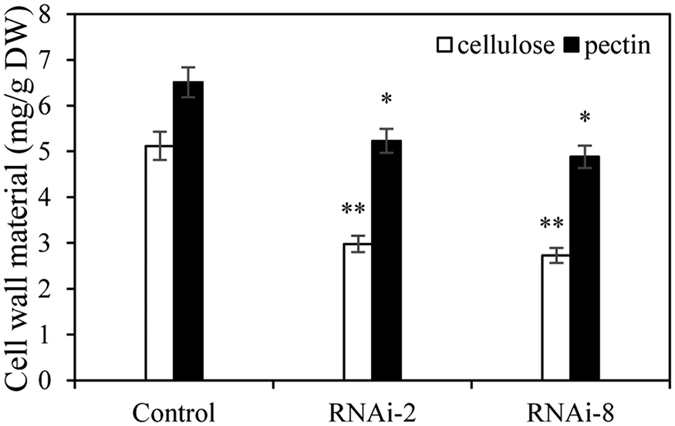
Comparison of cell wall composition of the control plants and transgenic plants. The content of cellulose and pectin in cell wall prepared from the control plants and transgenic plants (RNAi-2, RNAi-8). Results are significantly different from control under the same treatment conditions (Values are mean ± SE, *n* = 3, *P < *0.05*, **P < *0.01*).

It showed the cellulose and pectin content of the two transgenic lines (RNAi-2 and RNAi-8) and the control plants (Fig. 8). The result showed adout 40% decline in the cellulose content and about 19% reduction in the pectin content of the RNAi plants with that in the control plants. There were significant differences (P < 0.05) in cell wall materials between *CesA* T3 plants and control plants. It implied that the hairpin structure could affect cellulose biosynthesis.

### Proline and soluble sugar contents in the RNAi plants

**Figure 9:**
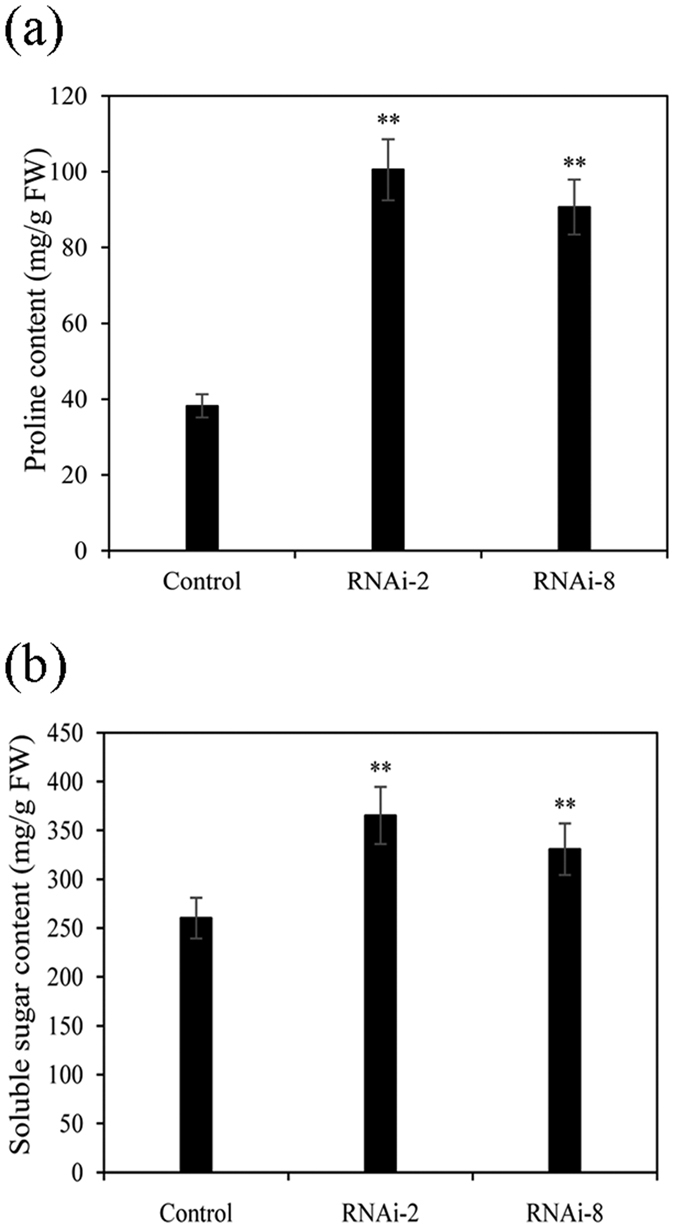
Content of proline (**a**) and soluble sugars (**b**) in RNAi and control plants under normal culture conditions. Values represent the mean ± SE from three independent experiments.

Accumulation of proline and soluble sugar is often related to plant adaptation to environmental stresses. Then in order to investigate the correlation of cellulose synthesis and plant physiological characters, proline and soluble sugar contents in T3 and control plants were measured under normal conditions. The two transgenic lines (RNAi-2, RNAi-8) respectively accumulated approximately 3 times higher proline contents than the control plants (Fig. 9a). At the same time, we found that the soluble sugar content of RNAi lines was higher (P < 0.01) than that in control plants (Fig. 9b).

### RNAi plants has higher salt resistance capability

**Figure 10:**
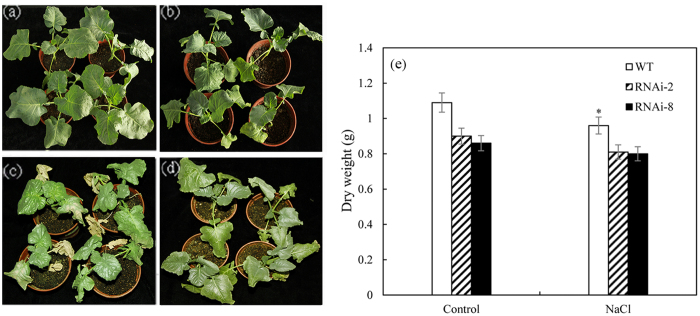
The RNAi plants are more NaCl tolerant than control plants. (**a**) Control plants without treatment. (**b**) The RNAi plants without treatment, RNAi-2 in left, RNAi-8 in right. (**c**) Control plants are bleached with 250 mM NaCl treatment for three weeks. (**d**) After 250 mM NaCl treatment for three weeks, there is no obvious change in RNAi transgenic plants. (**e**) Analysis of dry weight for control and transgenic plants under the NaCl treatment. Results are significantly different from control under the same treatment conditions (Values are mean ± SE, *n* = 3, *P < 0.05, **P < *0.01*).

In normal conditions, compared with control plants, the T3 of RNAi plants were dwarf phenotype with smaller dark green leaves (Fig. 10a and b). Under 250 mM NaCl treatment, the leaves of T3 were still green with thick waxy on the surface (Fig. 10d) whereas the control plants became bleached (Fig. 10c). Moreover under the NaCl treatment, the dry weight of control plants significant reduced relative to that of RNAi plants (Fig. 10e).

**Figure 11:**
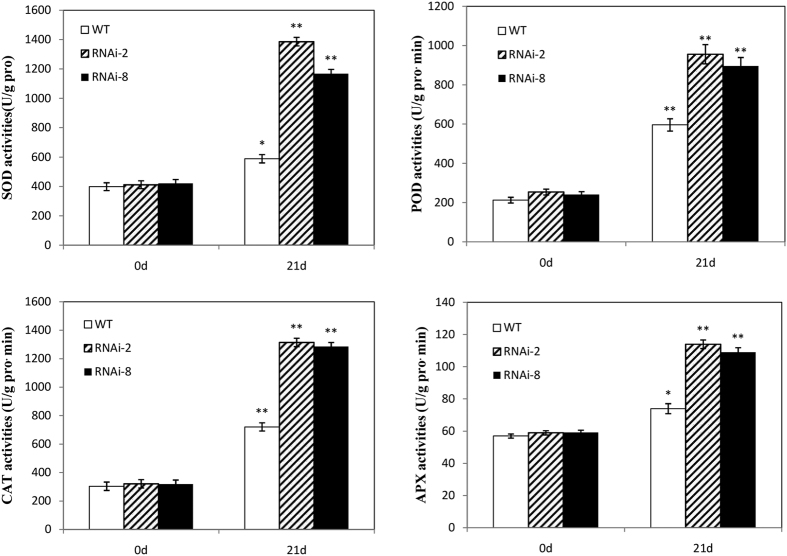
The measurement of superoxidedismutase (SOD) (**a**), peroxidase (POD) (**b**), catalase (CAT) (**c**) and ascorbateperoxidase (APX) (**d**) activities in broccoli plants under Salt treatment. Values are mean ± SE, *n* = 3, *P < 0.05, **P < 0*.01.*

Higher plants have developed an antioxidant defense system that includes the antioxidant enzymes SOD, POD, CAT, and APX to deal with adversity stress^44^. The enzymatic activity analysis of antioxidant system was conducted in transgenic and control broccoli under the NaCl treatment (Fig. 11). After 3 weeks of NaCl treatment, the SOD activity of RNAi plants was about 3-fold higher than that of control plants (Fig. 11a). The activities of POD, CAT and APX of RNAi lines showed similar trends (Fig. 11b, c and d).

### BoiCesA affects the expression of genes related to salt tolerance

**Figure 12:**
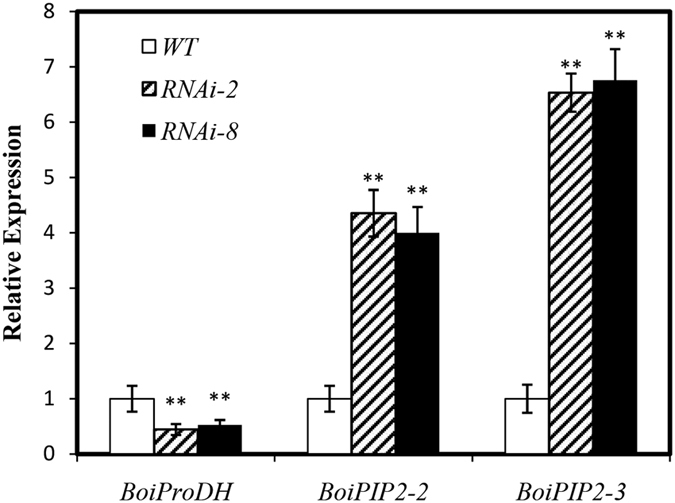
qRT-PCR of three genes differentially expressed in the WT and T3 of RNAi plants. Values are mean ± SE, *n* = 3, *P < 0.05, **P < *0.01.*

To further investigate the NaCl resistant mechanism of T3 of RNAi plants, we analyzed the expression of genes related to salt tolerance. Due to the lack of broccoli genome information, this brings some difficulties in our study. Based on the results of previous studies^45^,^46^,^47^, we found that *BoiProDH, BoiPIP2;2* and *BoiPIP;-3* genes are associated with the salt tolerance of plants (Fig. 12). The expression level of *BoiProDH* was significantly reduced in T3 of RNAi plants, it was about 0.5 time that of WT, while the expressions level of *BoiPIP2;2* and *BoiPIP2;3* up-regulated in T3 of RNAi plants, it were 6–7 times than those of WT, these results might explain the altered salt tolerance of T3 of RNAi plants.

## Discussion

The function of the cDNA corresponding to putative cellulose synthase gene from *Brassica oleracea* L. was analyzed by RNAi. In our study, in attempt to verify the function of the given *BoiCesA* gene, we constructed the special RNAi vector using the specific region of *CesA* gene which could be the basis of the multiple alignments. Based on the alignments for *CesA* gene from rice previously described, the phylogenetic relationship resulting from the analysis whether based on the alignments from complete amino acid sequences or the hypervariable region (HVR) sequences was the same, and the sub-class identify was primarily defined by the HVR. The sequence in this region does not vary among members of the same sub-class, these experiments already considered that the region was termed ‘class-specific region (CSR)’^48^. Hence, we chose the cDNA fragment located in the HVR region to inhibit the specific *BoiCesA* gene exclusively (see domain structure for plant *CesA* in Fig. 1a). It could void the lethal phenotype if several endogenous *CesA* genes were silenced by interference of the homologous region of all *CesA* genes; this phenomenon had been indicated^22^.

On the other hand, examination of the leaves from the T3 of RNAi plants by light and electron microscopy indicated extensive reduction of cell expansion in vascular bundles, particularly on their abaxial surface, the wider stomatal aperture and the changes of chloroplast ultrastructure due to the down-regulation of *BoiCesA* gene.

Previous theory has confirmed that microtubules and actin filaments form highly organized arrays in stomatal cells that play key roles in the morphogenesis of stomatal complexes^49^. Moreover, the cellulose fibrils and microtubules as well as actin filaments are radially distributed in guard cells^50^. The depositing cellulose microfibril affects the pattern of local wall thickenings and the mechanical properties of the walls of stomatal cells, thus regulates accurately their shape^49^. In our study, the modulation of cellulose content caused by the RNAi-induced silencing of the specific *BoiCesA* gene might be the reason of the wider stomatal aperture compared with the control, or, the reduction of the depositing cellulose micro-fibrils weakened support to the guard cells’ shape. In addition, we also observed the degradation of starch and the appearance of numerous osmiophilic globules in chloroplasts. The ultrastructural changes of leaves have been reported in wheat and corn^51^. The chloroplasts in sugarcane cultivar YT57/423 were collapsed, presenting many osmiophilic granules^52^. In addition under drought stress conditions, the chloroplasts’ lengths decreased and their widths increased, rendering them round in shape. Drought stress also significantly changed the internal structure of the chloroplasts. Membrane systems were damaged, starch grains disappeared, the chloroplasts became deformed and vacuolized^53^. Therefore, under stress condition, in chloroplasts the rapid degradation of starch and soluble sugars accumulation were occurred. Previous studies showed that drought stress can improve the content of some sugars, which may be suppressed some enzyme activities related to cellulose synthesis^54^, the *leaf wilting2* mutants which are new alleles of the *AtCesA8/IRX1* gene revealed that cellulose synthesis is important for stress responses containing drought induction of gene expression^55^. Our results suggest that regulation cell wall cellulose synthesis are significant influencing factors in polysaccharide metabolism and adaptations of plants to salt stress.

We know that the proline and soluble sugar are important osmotic protective substance that involved in osmotic adjustment^56^. It reported that proline acted as a compatible solute in the cytoplasm. The accumulation of proline can stabilize the macromolecules^57^,^58^. A central role of soluble sugars depends not only on their direct involvement in the synthesis of other compounds and energy provision, but also on stabilization of membranes^59^. The increment of soluble sugar and proline content associated with reductions in cellulose confirmed that the cell wall could not only perceive, but also adjust for physical changes in its structure.

Under the biotic and abiotic stress, the reduction of cellulose content was observed^60^. In the experiment the cellulose synthesis is important for NaCl stresses response. Compared with control plants, the T3 of RNAi plants were more tolerant to NaCl treatment. Cellulose is the main load-bearing component of cell wall. The change of cellulose content often generates obvious consequences either compensatory or integrity responses. For example, plants may be made more tolerant to stresses as drought, salt and osmotic stress by mutations at *AtCESA8* gene which encodes a subunit of a cellulose synthesis complex^55^. The *eli1* mutants of *CESA3* in Arabidopsis thaliana cause reduced cellulose synthesis, activating defense response through jasmonate and ethylene signaling pathways^61^. Those researches indicate that this is probably a general consequence of *CESA* loss-of- function.

In this study, we found the obvious starch degradation and soluble sugar accumulation in RNAi plants. It is possible that starchcould be converted into soluble sugar, which might resulted in the increase of soluble sugar content, and enhanced the salt tolerance ability of plants. On the other hand, knockdown of a cellulose synthase gene *BoiCesA* inhibited the synthesis of cellulose, might lead to the substrate of cellulose synthesis into other metabolic pathways, resulted increased soluble sugar content, which is involved in the intracellular osmotic potential. These inferences are still pending for further experimental verification.

Proline accumulation is a common response to osmotic stress in many plants. Proline dehydrogenase (ProDH) is a key enzyme that catalyzes the degradation of proline in the mitochondria^45^. Our previous results proved that it was effective to increase the accumulation of free proline by silencing *BoiProDH* gene under salt stress^46^. We found that the *BoiProDH* expression in RNAi plants was significantly decreased, which might reveal the proline accumulation in RNAi plants.

Aquaporins (AQPs), which play a regulatory role in cellular water transport also called membrane protein family MIP (major intrinsic protein). The function of aquaporin proteinabundant, such as water transport, osmotic adjustment, abiotic stress response. Our previous study confirmed that SlPIPs through improving plant water content and maintaining osmotic balanceto improve the ability of tomato drought resistance^62^. *BoiPIP2s* plays important roles in the response of broccoli to salinity^47^. The expressions of *BoiPIP2;2* and *BoiPIP2;3* in T3 of RNAi plants were significantly higher than in WT plants, this suggested that the excessive expression of genes related to salt tolerance enhanced the salt tolerance of RNAi plants.

Cellulose content has an influence on the vegetable quality and resistance. *BoiCesA* may play a crucial role in the control of cellulose biosynthesis, and it is the first report of the *CesA* gene cloned from broccoli, according to the genetic relationship of *CesA* genes in different plants. Based on the different plant *CesA* genes dendrogram, we predict that the *BoiCesA* clone is a member of *CesA* family and the *BoiCesA* clone may share functional similarity with the *Arabidopsis* genes in the same cluster. Without the function of the *CesA1, CesA2, CesA3* and *CesA6* genes, the cellulose of primary wall are difficultly biosynthesized from mutants^17^,^23^,^63^,^18^. So we suggest that the *BoiCesA* clone may play a role in primary cell wall biosynthesis.

## Additional Information

**How to cite this article:** Li, S. *et al*. Knockdown of a cellulose synthase gene *BoiCesA* affects the leaf anatomy, cellulose content and salt tolerance in broccoli. *Sci. Rep.* **7**, 41397; doi: 10.1038/srep41397 (2017).

**Publisher's note:** Springer Nature remains neutral with regard to jurisdictional claims in published maps and institutional affiliations.

## Supplementary Material

Supplementary Information
