# Supplementary material for: Knockdown of a cellulose synthase gene BoiCesA affects the leaf anatomy, cellulose content and salt tolerance in broccoli
Source: Sci Rep. 2017 Feb 7;7:41397. doi: 10.1038/srep41397 (PMC5294630; doi:10.1038/srep41397)
Supplement: Supplementary Information [file srep41397-s1.doc]

**Knockdown of a cellulose synthase gene *BoiCesA* affects the leaf anatomy, cellulose content and osmotic and salt tolerance in broccoli**

Shuangtao Li^1¶^ , Ying Wang^1, 2¶^, Fengfeng Xu^1 ¶^ , Lei Zhang^1^, Mengyun Liu^1^, Peng Lin^1^,

Shuxin Ren^3^, Rui Ma^4^, Yang-Dong Guo^1^*

^1^ College of Horticulture, China Agricultural University, 100193, Beijing, China;

^2^ Horticulture Research Institute, Shanghai Academy Agricultural Sciences,Shanghai 201403, China;

^3^ School of Agriculture, Virginia State University, PO Box 9061, Petersburg, VA23806, USA;

^4^ Agro-Biotechnology Research Institute, Jilin Academy of Agricultural Sciences, Changchun 130033, China.

^¶^ These authors contributed equally to this work.

*Authors for correspondence:


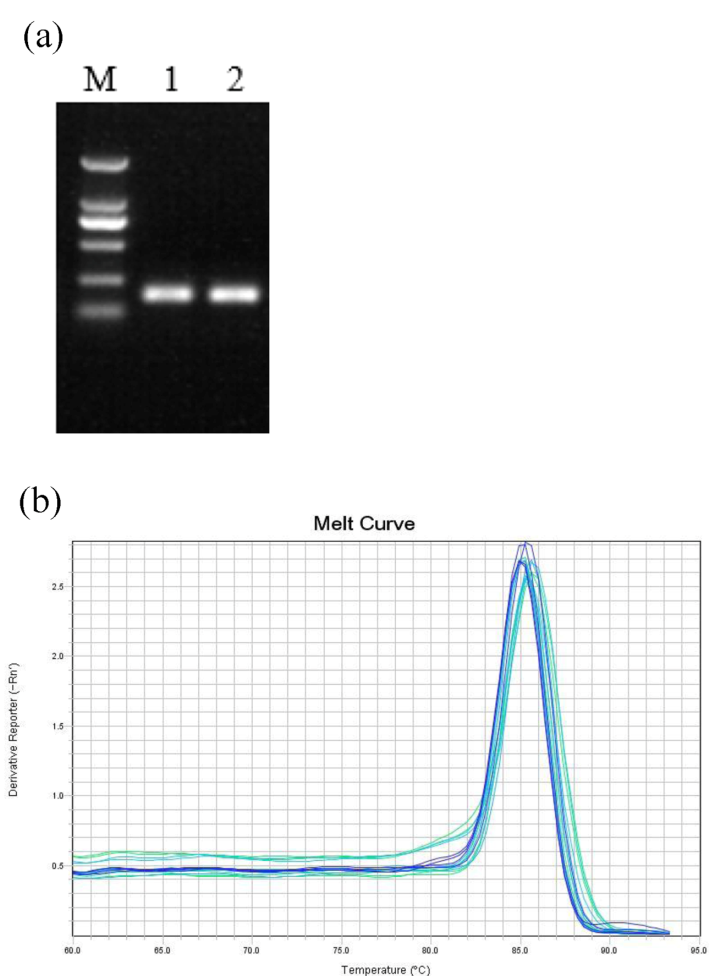


# Supporting information

**S1 Fig. The specific of primer pairs was checked.**

(a) Identification of the specific primers by PCR.

(b) The melt curve of quantitative real-time PCR.
